# Biomolecular evidence for changing millet reliance in Late Bronze Age central Germany

**DOI:** 10.1038/s41598-024-54824-0

**Published:** 2024-02-22

**Authors:** Eleftheria Orfanou, Barbara Zach, Adam B. Rohrlach, Florian N. Schneider, Enrico Paust, Mary Lucas, Taylor Hermes, Jana Ilgner, Erin Scott, Peter Ettel, Wolfgang Haak, Robert Spengler, Patrick Roberts

**Affiliations:** 1 Department of Archaeogenetics, Max Planck Institute for Evolutionary Anthropology, 04103 Leipzig, Germany; 2 Department of Archaeology, Max Planck Institute of Geoanthropology, 07745 Jena, Germany; 3 Chair of Pre- and Protohistoric Archaeology, Friedrich-Schiller University Jena, 07743 Jena, Germany; 4 Domestication and Anthropogenic Evolution Research Group, Max Planck Institute of Geoanthropology, 07745 Jena, Germany; 5 School of Computer and Mathematical Sciences, University of Adelaide, Adelaide, 5005 Australia; 6 Arctic University Museum of Norway, UiT-the Arctic University of Norway, Lars Thørings Veg 10, 9006 Tromsø, Norway; 7 Department of Anthropology, University of Arkansas, Fayetteville, 72701 USA; 8 isoTROPIC Research Group, Max Planck Institute of Geoanthropology, 07745 Jena, Germany; 9 Institut Für Ur- Und Frühgeschichte, Philosophische Fakultät, Universität Zu Köln, Cologne, Germany

**Keywords:** Archaeology, Palaeoecology, Stable isotope analysis

## Abstract

The Bronze Age of Central Europe was a period of major social, economic, political and ideological change. The arrival of millet is often seen as part of wider Bronze Age connectivity, yet understanding of the subsistence regimes underpinning this dynamic period remains poor for this region, in large part due to a dominance of cremation funerary rites, which hinder biomolecular studies. Here, we apply stable isotope analysis, radiocarbon dating and archaeobotanical analysis to two Late Bronze Age (LBA) sites, Esperstedt and Kuckenburg, in central Germany, where human remains were inhumed rather than cremated. We find that people buried at these sites did not consume millet before the Middle Bronze Age (MBA) (ca. 1600 BCE). However, by the early LBA (ca. 1300–1050 BCE) people consumed millet, often in substantial quantities. This consumption appears to have subsequently diminished or ceased around 1050–800 BCE, despite charred millet grains still being found in the archaeological deposits from this period. The arrival of millet in this region, followed by a surge in consumption spanning two centuries, indicates a complex interplay of cultural and economic factors, as well as a potential use of millet to buffer changes in aridity in a region increasingly prone to crop failure in the face of climate change today.

## Introduction

The Bronze Age in Central Europe (ca. 2300 to 800 BCE) is characterized by increasing social complexity and cultural connectivity, as well as social, ideological, political, and economic change^1–3^. During this period, metallurgy became well-established and the use of bronze and other metals became widespread^4^. Since these metals were not always locally available, extensive trade networks were established^5^. Alongside these processes, during the Late Bronze Age (LBA) (ca. 1300 to 800 BCE), there was a change in burial practices, from inhumation to cremation, likely marking a shift in ideology and/or in practical considerations^6^. Despite these broader social changes, our understanding of agricultural developments over this time period remains limited for many parts of Central Europe.

The environments of temperate Europe are dominated by wild C_3_ vegetation, from forests and woodlands to temperate grasslands^7,8^. The crops that arrived in this part of the world during the Neolithic (ca. 5500 to 2300 BCE), including wheat and barley, were also C_3_ crops. Such plants are seen as being well-adapted to the wet and cool climate^9–11^. Archaeobotanical studies have demonstrated that broomcorn millet (*Panicum miliaceum*) dispersed into Europe from northeast China by the middle of the 2nd millennium BCE^12–16^, adding to the established food production systems based on wild and domesticated (e.g., barley, wheat) plants and animals. Many archaeologists have pointed out that the drought resistance and relative ease of cultivation of this crop may have facilitated its rapid dispersal and adoption across Eurasia^17,18^. Millet is a C_4_ plant, and therefore demonstrates an advantage in arid and warm conditions. Furthermore, millet has a short growing period (around three months in Central Europe), high yield, and is suitable for long-term storage, making it an ideal crop for diverse socioeconomic systems and dynamic environmental conditions^18–20^.

In this paper, we take an interdisciplinary approach, looking at human and animal diets, as well as botanical evidence, in order to determine the extent of the incorporation of this crop into subsistence strategies and its role within socioeconomic adaptations more broadly. Stable isotope analysis of human remains, in particular, enables direct assessment of individual and group reliance on C_3_ versus C_4_ plants, as well as the degree of meat and aquatic resource consumption^21–23^. Stable carbon isotope values (δ^13^C) vary in plants following different (C_3_ and C_4_) photosynthetic pathways, since they fractionate the two stable isotopes of carbon differently. This leads to distinct, non-overlapping ranges of δ^13^C values. C_3_ plants exhibit a range of − 35 to − 20‰^21,24^, while C_4_ plants range from − 14 to − 9‰^25,26^. These differences in δ^13^C values are reflected in the tissues of consumers, but with a trophic effect of 1–2‰, allowing some estimation of the importance of these different resources in the food chain^27,28^. On the other hand, stable nitrogen isotope ratios (δ^15^Ν) are associated with feeding behaviour (e.g., trophic level) and environmental factors that shape the organism’s physiology^29,30^.

In Europe, the early use of millet has been demonstrated through stable isotope analysis in certain regions and time periods. Studies have shown millet use during the MBA and LBA in Poland and western Ukraine^31^, as well as in northern Germany^32^, Croatia^33^, Iberia^34^, Italy^35^, and Greece^36^. Stable isotope studies have also been used to trace the routes of millet dispersal across Central Eurasia^16,37–39^. Most of the above studies focus on the earlier phase of the LBA (ca. 1300–1050 BCE), however, with few results (e.g.,^31^) concerning the later phase (ca. 1050–800 BCE). Millet seeds have been identified in various LBA sites in central Germany^40^, which could indicate the presence of the crop in the region during that period. However, there is a lack of stable isotope studies in central Germany during the LBA, in large part due to the shift to the mortuary practice of cremation, since collagen does not survive the high temperatures generated during the cremation process, making past diet reconstruction impossible^41^.

**Figure 1:**
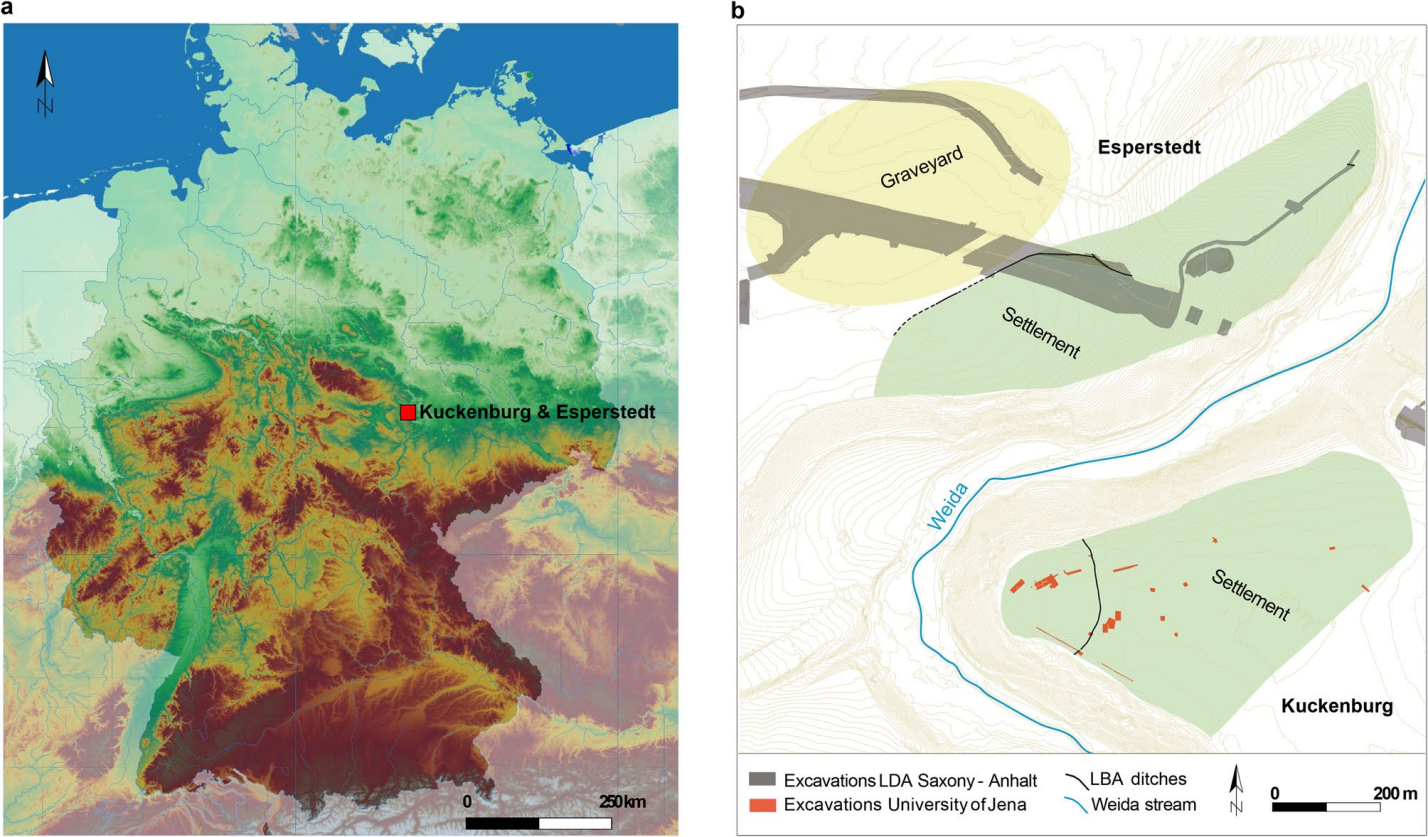
Location of archaeological sites. (**a**) Location of Kuckenburg and Esperstedt within Saxony—Anhalt in central Germany. (**b**) Excavation map of the two archaeological sites, demonstrating the hilltop settlement of Kuckenburg and the settlement and graveyard of Esperstedt. Maps of (**a**) and (**b**) were generated using QGIS software (v3.12.2-București https://qgis.org/). The original base maps were extracted from (**a**) the SRTM Data web site (https://srtm.csi.cgiar.org/srtmdata/) and (**b**) © GeoBasis-DE / LVerm-Geo ST, 2017, 167 (www.govdata.de/dl-de/by-2-0).

In this study, we apply stable isotope analysis to human (*n* = 53) and animal (*n* = 22) remains from the archaeological sites of Esperstedt and Kuckenburg in Saxony-Anhalt, central Germany (Fig. 1). New radiocarbon dates and archaeobotanical information is used to contextualise the data. Esperstedt and Kuckenburg are both multi-period sites with non-continuous occupations spanning from the mid-4th millennium BCE to the early Middle Ages (eighth—eleventh century AD). Here we focus on the LBA (phase Bz D (1300–1200 BCE), Ha A (1200–1050 BCE) and Ha B (1050–800 BCE)) occupation periods, which for the purpose of this study are divided into early LBA (Bz D and Ha A) and late LBA (Ha B), and compare these with previous periods in order to study changes through time. Esperstedt comprises a LBA graveyard and a LBA settlement, whereas Kuckenburg is a LBA hilltop settlement. These two sites are located in close proximity to each other (< 2 km) (Fig. 1). The LBA individuals from the sites belong to the Unstrut cultural group (1325–750 BCE**)**, named after the main river flowing by their settlement. A series of archaeological features distinguish the Kuckenburg/Esperstedt micro-region as particularly favourable for osteoarchaeological and biomolecular research. Notably, both sites have yielded inhumation burials, something also characteristic in the Thuringian Basin ("Thüringer Becken") but very rare for the LBA in Central Europe more generally, which is primarily characterized by cremation burials^42^. Not only that, but these inhumations are also found in settlement contexts. Although settlement burials have also been identified in the Saale-Unstrut-Triasland in the southern part of Saxony-Anhalt^43^**,** the fact that there are two different kinds of settlements with human depositions (i.e., a hilltop settlement—Kuckenburg and an open settlement—Esperstedt) and a graveyard (Esperstedt) makes the Kuckenburg/Esperstedt micro-region almost unique in the LBA of Central Europe. Ongoing excavations at Kuckenburg and further research on the materials from Esperstedt are expected to contribute to our understanding of the factors behind the characteristics of this micro-region.

## Results

### ^14^C dating

Out of the 53 human individuals from Kuckenburg and Esperstedt, 45 were directly dated and 39 have been attributed to the Bronze Age (Supplementary Table 1 (S1)). More specifically, five of these dates range between 2293 and 1751 cal. BCE, corresponding to the EBA and the Únětice culture. Additionally, 13 dates fall between 1406 and 1055 cal. BCE, representing the early phase of the LBA. The remaining 22 dates span from 1041 to 766 cal. BCE, aligning with the later phase of the LBA. Of the remaining five individuals, two were dated to the Middle Neolithic (MN) (3640–3022 cal. BCE) and three to the Final Neolithic (FN) (2875–2235 cal. BCE). For the eight directly dated animal remains, the dates obtained fall between 1434 and 766 cal. BCE, providing further support for the archaeological dating of these features as belonging to the LBA. Finally, the five dated millet seeds range from 1366 to 820 cal. BCE. Among these, three charred millet seeds date back to the early LBA, with dates ranging from 1366 to 1054 cal. BCE. The remaining two charred millet seeds can be attributed to the later phase of the LBA, with dates falling between 1006 and 820 cal. BCE.

### δ^13^C and δ^15^N diet isotope analysis of bone collagen and δ^13^C from tooth enamel

**Figure 2:**
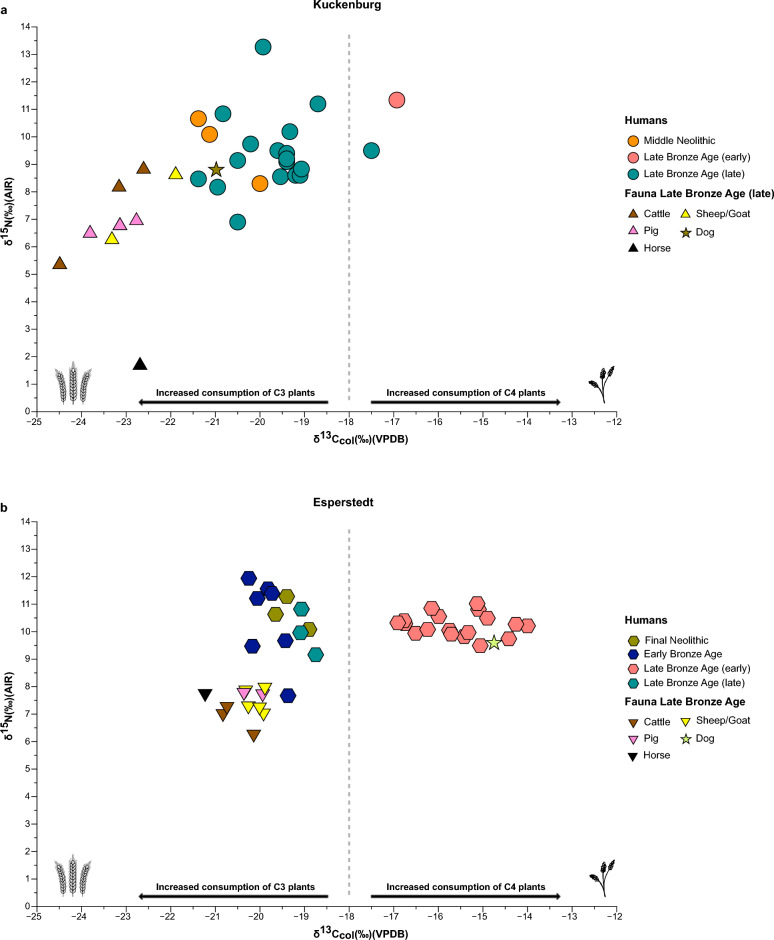
Comparison of δ^13^C and δ^15^N values from bone collagen for ancient individuals and fauna from Kuckenburg and Esperstedt. The dashed line represents millet’s “threshold” of δ^13^C = −18 ‰^14,47^. (**a**) δ^13^C and δ^15^N values for Kuckenburg individuals and fauna. (**b**) δ^13^C and δ^15^N values for Esperstedt individuals and fauna. Wheat icon (left) was created by Oleksandr Panasovskyi from Noun Project (CC BY 3.0), https://thenounproject.com/browse/icons/term/wheat/. Millet icon (right) was created by Roman from Noun Project (CC BY 3.0), https://thenounproject.com/browse/icons/term/millet/.

We were able to successfully extract good-quality collagen from all samples, as indicated by their C:N elemental ratio values, weight percent of carbon (% C), nitrogen (% N), and percent collagen yield^44–46^. The δ^13^C and δ^15^N measurements for the analysed individuals are provided in Supplementary Table 2 (S2) and shown in Fig. 2.

At Kuckenburg, the 22 analysed individuals had δ^13^C collagen values ranging between –21.4‰ and –16.9‰ (mean = − 19.7‰ ± 1.1), while the range of their δ^15^N values was from 6.9 to 13.3 ‰ (mean = 9.5 ‰ ± 1.4) (Fig. 2a). The δ^13^C and δ^15^N values for the six herbivore samples (cattle, sheep/goat, and horse) range from − 24.5 to − 21.9‰ (mean = − 23‰ ± 0.9) and from 1.7 to 8.8 ‰ (mean = 6.5‰ ± 2.7), respectively. The three omnivore samples (pig) have δ^13^C and δ^15^N values ranging from − 23.8 to − 22.8 ‰ (mean = − 23.2‰ ± 0.5) and from 6.5 to 7.0‰ (mean = 6.7‰ ± 0.2), respectively. The dog had a δ^13^C value of − 21.0‰ and a δ^15^N value of 8.8‰ (Fig. 2a).

At Esperstedt, the 31 analysed individuals had δ^13^C values ranging between –20.3 ‰ and –14.0 ‰ (mean = − 17.2 ‰ ± 2.1), with δ^15^N values ranging from 7.7 ‰ to 11.9 ‰ (mean = 10.3 ‰ ± 0.8) (Fig. 2b). The δ^13^C and δ^15^N measurements for the 9 herbivore samples (cattle, sheep/goat, and horse) range from − 21.2 to –19.9 ‰ (mean = − 20.4 ‰ ± 0.5) and from 6.3 to 8 ‰ (mean = 7.3 ‰ ± 0.5), respectively. The two omnivore samples (pig) have δ^13^C and δ^15^N measurements ranging from − 20.4 to − 19.9 ‰ (mean = − 20.2 ‰ ± 0.3) and from 7.7 to 7.8 ‰ (mean = 7.8 ‰ ± 0.0), respectively. The dog had a δ^13^C value of −14.8 ‰ and a δ^15^N value of 9.6 ‰ (Fig. 2b).

In Fig. 2a,b, we observe a clear shift of the δ^13^C values of the ancient individuals between time periods. Individuals from the early phase of the LBA (*n* = 20) show values from − 19.4 to − 14.0 ‰ (mean = − 15.8 ‰ ± 1.2), while individuals from all the other time periods (*n* = 33) show values from − 21.4 to − 17.5 ‰ (mean = − 19.7 ‰ ± 0.8). The δ^15^N values between these time periods range from 7.7 to 11.3 ‰ (mean = 10.2 ‰ ± 0.7) in the early phase of the LBA to δ^15^N values from 6.9 to 13.3 ‰ (mean = 9.9 ‰ ± 1.3) for all the other periods.

We conducted Kruskal–Wallis rank sum tests^48^ to examine if the differences in δ^13^C and δ^15^N values between the time periods are statistically significant. The tests revealed a significant difference in both δ^13^C (Kruskal–Wallis chi-squared = 36.871, df = 4, *p* = 1.915e-07) and δ^15^N (Kruskal–Wallis chi-squared = 16.353, df = 4, *p* = 0.00258) values, between time periods. In order to examine where the differences lie, we performed post hoc Dunn's tests to examine pairwise comparisons of δ^13^C and δ^15^N values among the time periods and used the Bonferroni adjustment to correct for multiple comparisons^49^. The results of the Dunn's tests for the δ^13^C values indicated significant differences between the following pairs (adjusted p-values): EBA—early LBA (Z = −3.655, *p* = 0.002), early LBA—late LBA (Z = 4.843, *p* = 0.00001), early LBA—FN (Z = 3.019, *p* = 0.025), and early LBA—MN (Z = 3.914, *p* = 0.001). The results of Dunn's tests for the δ^15^N values indicated significant differences between the following pairs (adjusted p-values): EBA—late LBA (Z = 3.709, *p* = 0.002). Detailed results can be found in Supplementary Table 3 (S3).

A correlation test using the Spearman coefficient indicated no significant correlation between δ^13^C and δ^15^N for the late LBA individuals from Kuckenburg (ρ = 0.25, *p* = 0.316). Furthermore, a correlation test using the Spearman coefficient indicated no significant correlation between δ^13^C and δ^15^N in the early LBA individuals from Esperstedt (ρ = 0.056, *p* = 0.817) (Supplementary Table 3 (S3)).

**Figure 3:**
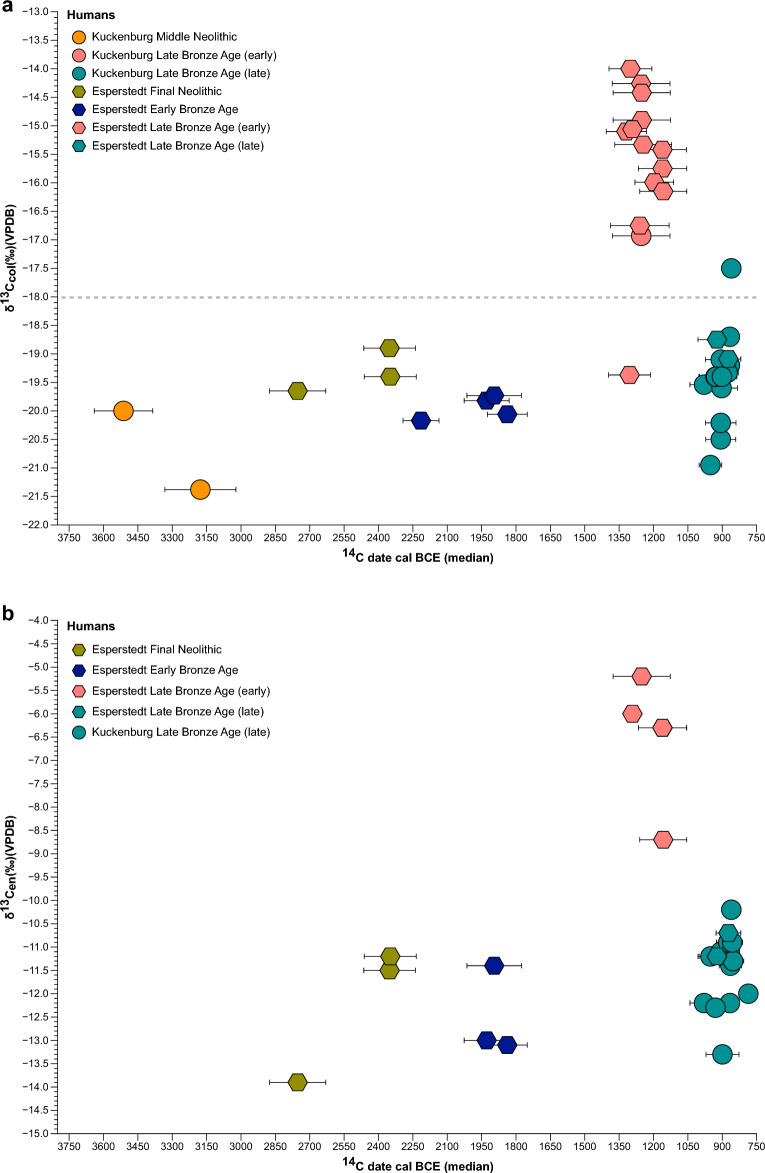
Comparison of δ^13^C human values from bone collagen and tooth enamel over time. The δ^13^C values are plotted against ^14^C calibrated dates (median) for ancient individuals from Kuckenburg and Esperstedt over/across a time transect including MN, FN, EBA, and LBA individuals. (**a**) δ^13^C values from bone collagen plotted against ^14^C calibrated dates (median). The dashed line represents a “threshold” of −18 ‰^14,47^. (**b**) δ^13^C values from tooth enamel plotted against ^14^C calibrated dates (median).

Following previous isotope studies in the region (e.g.,^14,47^), we set a δ^13^C collagen value of − 18 ‰ as a threshold for increased consumption of C_4_ plants in Europe (Figs. 2 and 3). Consequently, values higher than − 18 ‰ indicate the consumption of a mixed C_3_/C_4_ diet and a medium consumption of millet, while values higher than − 12 ‰ correspond to a diet predominantly based on C_4_ plants^14,31^. Figure 3 depicts the differences in δ^13^C values (from collagen and tooth enamel) among time periods based on direct radiocarbon dates. Here, we observe that individuals from the MN, FN, EBA and late phase of LBA were consuming a C_3_ based diet, while individuals from the early phase of the LBA were consuming C_4_ plants as well, as indicated by their δ^13^C values.

We also conducted δ^13^C_en_ on the enamel of both our human individuals and available fauna to complement the protein-biased bone collagen values with δ^13^C_en_ measurements of the 'whole diet'^28^. This approach allows us to gain a more comprehensive understanding of the dietary habits of the ancient population. Teeth predominantly reflect dietary patterns during childhood and adolescence, whereas bones offer insights into dietary practices approximately during the last 10 to 25 years (depending on specific bone remodelling rate) preceding an individual's death^50–52^. By considering both dental and bone isotopic data, we can also better discern the long-term dietary trends and behaviours of the studied individuals.

The δ^13^C_en_ values of the animal samples (*n* = 6) from the later phase of the LBA in Kuckenburg vary between − 17.2 and − 9.5 ‰ (mean = − 12.9 ‰ ± 3). Specifically, the δ^13^C_en_ values for sheep/goats (*n* = 3) range from − 15.7 to − 11.2 ‰ (mean = − 13.2‰ ± 2.3). For cattle (*n* = 2), we observe a range from − 17.2 to − 10.9‰ (mean = − 14 ‰ ± 4.5). One hamster sample has a δ^13^C value of − 9.5 ‰. At Kuckenburg, the analysed human individuals (*n* = 17) had δ^13^C_en_ values ranging between − 13.3 ‰ and − 10.2 ‰ (mean = − 11.6 ‰ ± 0.8). At Esperstedt, the analysed individuals (*n* = 16) had δ^13^C_en_ values ranging between − 13.9 ‰ and − 5.2 ‰ (mean = − 10.2 ‰ ± 2.9). All available δ^13^C_en_ measurements from tooth enamel of the individuals analysed are shown in Supplementary Table 2 (S2) and plotted in Fig. 3b.

We also conducted Kruskal–Wallis rank sum tests to examine if the differences in δ^13^C human enamel values between the time periods are statistically significant. The tests revealed a significant difference in δ^13^C (Kruskal–Wallis chi-squared = 15.532, df = 3, *p* = 0.001) between time periods. The results of the Dunn's tests for the δ^13^C values indicated again significant differences between the following pairs (adjusted *p*-values): EBA—early LBA (Z = −3.601, *p* = 0.002), early LBA—late LBA (Z = 2.957, *p* = 0.02), and early LBA—FN (Z = 2.713, *p* = 0.04) (Supplementary Table 3 (S3)).

### Archaeobotany

More than 200 sediment samples were collected for macrobotanical investigations during the excavations since 2007. Of the 87 processed samples, 19 contain grains from millet. For radiocarbon dating, seeds of *Panicum miliaceum* from five of these samples were selected coming from five different excavation pits; two of which bisected a ditch (feature 1/2019). The millet grains from those two pits are around 200 to 300 years younger (ranging between 1006 and 820 cal. BCE) than those of the other samples (ranging between 1366 and 1054 cal. BCE, Supplementary Table 1 (S1)). In one of the two pits, which also held a burial, a sample of 2.3 L contained only a single millet grain together with three einkorn grains (*Triticum monococcum*). The other sample (5.1 L), held a millet grain that we dated to be slightly older. The latter sample also contained chaff from three more cereal species, hulled barley (*Hordeum vulgare* var *vulgare*), einkorn, and spelt (*Triticum aestivum* ssp. *spelta*), as well as lentils (*Lens culinaris*). Free-threshing bread wheat (*Triticum aestivum)* and emmer (*Triticum dicoccum*) only occur in one of the pits outside the ditch together again with barley, einkorn, spelt, and lentils. The only identified crop with oil-producing seeds is gold of pleasure (*Camelina sativa*), present in the sample from the pit with the burial and in one of the pits from outside the ditch. Broomcorn millet grains were discovered in sediments spanning the time period indicated by the radiocarbon data. Whether the difference in the spectrum of cereal species through time shows any modification must be determined through the ongoing research and analysis of more samples.

## Discussion

During the LBA in central Germany, changes occurred not only in burial customs and various aspects of daily life (e.g., intensified trade) but also in agricultural practices and culinary traditions^53^. In our study, we observe a shift from cultivating and consuming exclusively C_3_ plants, such as wheat and barley, to the inclusion of a new crop, broomcorn millet, in the agricultural repertoire. Specifically, at the site of Esperstedt, δ^13^C values higher than − 18 ‰ correspond to the earlier phase of the LBA (ca. 1300–1050 cal. BCE) and they are indicative of the inclusion of C_4_ plants in the diet^54^. At Kuckenburg, the same applies to one sampled individual (BEF.36) from the early phase of the LBA. The range of the human δ^15^N values is indicative of an omnivorous human diet and the human-domesticated fauna offset, Δ^15^N_h-f_ = 2.5 ‰, is slightly lower than the typical 3–5 ‰^51^.

At Kuckenburg, the following exceptions should be noted: One individual (KUC008) with a lower δ^15^N value of 6.9 ‰ might have consumed less animal protein in their diet compared to the other individuals. Meanwhile, the individual (KUC019) with the higher value δ^15^N of 13.3 ‰ is an infant and this value might be indicative of breastfeeding, a period when we would expect the infant's tissues to have elevated values^55^. The elevated δ^13^C values observed in the early LBA individuals could also possibly be interpreted as the consumption of freshwater fish, since they would have been available, as both sites are near the Weida stream (Fig. 1b). However, Eurasian lacustrine fish tend to have lower δ^13^C values than the observed values^56^. In addition, we did not observe a significant correlation between δ^13^C and δ^15^N values, as might be expected if changes in δ^13^C were linked to aquatic input. If people were consuming freshwater fish, we would expect their δ^15^N values to be much higher (δ^15^N > 12 ‰)^56,57^, and possibly find some fish bones in the soil (although this is rare in dry soils), which so far is not the case, despite using flotation devices during the excavation.

For reasons that are unclear, people buried at these two sites ceased millet consumption around 1050 BCE. Specifically, we observe that the δ^13^C range in the hilltop settlement of Kuckenburg during the late phase of the LBA is primarily indicative of a C_3_-based diet, with the exception of only one individual (KUC012) who seems to have consumed small amounts of millet (δ^13^C = − 17.5 ‰). The range of the δ^15^N values is consistent with a typical omnivorous human diet. Two individuals from the later phase of the LBA Esperstedt settlement site show lower values than − 18 ‰ (i.e., ESP036 = − 18.7 ‰ and ESP041 = − 19.1 ‰), which suggests the consumption of entirely C_3_ resources. A gradual phasing out of millet could potentially be supported by the individual KUC012. This individual may represent the transition from the consumption of millet to the cessation of this consumption, since these lower values could indicate dietary changes away from millet in the last few years before he died. A comparable trend of diminishing millet consumption was observed in Poland in the study of Pospieszny et al. (2021). Notably, the only late LBA individuals from Poland (mk83 and mk84) exhibited values suggestive of reduced millet intake (mk83: δ^13^C = − 17.8 ‰ and mk84: δ^13^C = − 17.6 ‰)^31^. Animals from both the early and late phases of the LBA at both sites do not appear to have been consuming millet or any other kind of C_4_ vegetation, except for a dog at Esperstedt showing a δ^13^C value of − 14.8 ‰. This finding is consistent with the premise that dogs typically share a diet similar to that of humans, often scavenging or being fed food scraps by their owners^58,59^. The δ^13^C range observed in the domesticated animals suggests their diet comprised wild C_3_ plants or fodder derived from C_3_ crops. The Δ^15^N_h-f_ offset in the human-domestic fauna, at 3 ‰, aligns with a typical predator–prey relationship^51^. No clear C_4_ signal is evident in the animals. However, a comparison between the animals from Esperstedt and Kuckenburg reveals a slightly different picture. We observe lower δ^13^C values in the Kuckenburg animals by about 3 ‰ (Fig. 2). This shift suggests that the animal fodder in Esperstedt may have included some millet, which would parallel the human diets at the site. Alternatively, it could signify that Esperstedt animals grazed on drier pastures than those from Kuckenburg, resulting in higher δ^13^C values within the C_3_ space, or wild C_4_ plants. At the same time, it is possible that herding practices for the Kuckenburg animals included graze in undergrowth from forested areas, which is typically characterized by lower δ^13^C values than open pastures^60,61^. However, given the apparent importance of millet at Esperstedt during the early LBA, it is plausible that millet fodder also played a role.

In the Mittelelbe-Saale region of central Germany, where the Esperstedt and Kuckenburg sites are located, millet has been discovered in sediments dated to the LBA in various forms, such as isolated seeds, charred grain lumps, and directly-dated mass findings^40^. The majority of the radiocarbon dates obtained from seeds fall within 1300–1100 BCE, with only one site providing evidence for a later occurrence (1000–800 BCE). A comprehensive diachronic study of the Mittelelbe-Saale region conducted by Münster and colleagues^62^ investigated the dietary habits of people across a span of 4000 years, ranging from the Early Neolithic to the Early Bronze Age (EBA) (ca. 5500–1550 BCE). This study also provided dietary insights for Esperstedt, and revealed consistent consumption of C_3_ plants throughout the examined chronology, while no consumption of C_4_ plants was identified. Nevertheless, the temporal scope of Münster et al.'s study only extends up to the EBA, leaving a temporal gap during the LBA. Our data therefore provide new insights into the tempo of millet adoption and, particularly, its importance to diets and human economies during the LBA period. This provides new insights into the variability of Central European dietary strategies at this time. It is less clear when the consumption of millet in the wider region started. From our dataset, the latest dates for an absence of millet consumption come from late EBA contexts, dating to ca. 1800–1750 BCE, while the first attested date of possible millet consumption is ca. 1400 BCE. We then observe a cessation of consumption of millet around 1050 BCE. This leaves a wide window of several centuries and thus a multitude of factors that may explain these shifts, such as changes in trade networks, population growth, changes in cultural preferences, and climate variability.

Although it might seem relatively short from the perspective of prehistory, 250 years of intensive millet consumption (i.e., 1300–1050 BCE) would have represented about 8–9 generations^63^,—a considerable time frame in the life of prehistoric communities. Food, beyond its nutritional role, serves as a nexus for numerous cultural and social processes, shaping identity, creating and maintaining collective memories, and influencing societal dynamics^64^. Eating habits sustain a sense of identity and community during challenging times and embody practical knowledge of how individuals interact with their environment^64,65^. Economic considerations, including availability, convenience, yield, access, and environmental conditions can influence the choice of crops and of agricultural practices. However, the acceptance or rejection of specific foods (or crops) by individuals or communities, is the result of the entanglement of economic with cultural and social factors (e.g., taste preferences, tradition, memory, emotions, social position, etc.)^64,66,67^.

The processes behind the spread of new foodstuffs are debated in archaeology, often focusing on demographic spread bringing new cultural and economic preferences versus active adoption by resident communities^68–71^. The archaeological situation of Kuckenburg and Esperstedt provides an interesting case in this regard. On the one hand, some cremations in the Esperstedt graveyard perhaps point to a possible influence from nearby cultural groups (for example the Saalemündungsgruppe), where cremations are the predominant burial form/type^72–74^. However, further research is necessary to assess the chronology of these cremations as well as any potential link to other communities. On the other hand, overall, Kuckenburg and Esperstedt show a number of unique features in terms of burials including inhumations in both open and hilltop settlement contexts, special treatment of isolated body parts (e.g., skull depositions) in some cases, and, in Esperstedt, an absence of the use of stone cists seen in the nearby Saalemündungsgruppe^72,74^ (see Supplementary Text 1 and 2 for more details).

Based on the existing archaeological contextual information, it is therefore likely that changes in crop use and economy reflect the local navigation of particular economic, cultural and environmental conditions. In particular, we propose that climatic fluctuations could also have impacted the availability and productivity of certain crops, shaping economic focus on millet in Central Europe, especially since millet is more resistant to periods of lower precipitation and provides a higher yield in unfavourable climatic conditions. While specific paleoclimatic records for the area of interest are lacking, evidence from the broader region supports the presence of climatic shifts. Pollen records from Germany indicate climate changes around 1500 BCE, which continued until approximately 500 BCE, characterized by decreasing precipitation, lower winter temperatures, and fluctuating summer temperatures^75^. The start of this arid period coincides with the time when millet consumption emerged in our dataset (ca. 1400 BCE). Additionally, a period of rapid climate change around 1450–450 BCE, characterized by polar cooling, tropical aridity, and major atmospheric circulation changes, has been identified in globally distributed paleoclimate records^76^. Moreover, the 3.2 ka BP event, which affected the Mediterranean region^77–79^, might have also affected Central Europe^78,80^. There is evidence from many regions in Europe (Ireland, Britain, Nordic area, Carpathian Basin, the Po Valley and the Aegean region) of a period of increased aridity between 1200 and 900 BCE. Increasing aridity and temperatures had started in certain areas (e.g., Pannonian plain, Po Valley) from 1400 BCE onwards^81–83^, peaking around 1250 BCE.

The sites discussed here are situated in the central German dry area ("Mitteldeutsches Trockengebiet") in the rain shadow of the Harz Mountains, with annual precipitation ranging from 460 to 550 mm, the lowest in Germany^40,84^, making conditions not only vulnerable to any changes in the length or intensity of the dry season, but also favourable for cultivating drought-tolerant crops like millet. If indeed there was a period of increased aridity in the area, the local population may have adapted to the new conditions and modified certain practices to ensure survival. Periods of increased aridity have been documented in Central Europe throughout history^85,86^ and more recently^87–89^, resulting in numerous challenges including diminished yields of crops with lower tolerance to such adverse conditions. Given the exceptional resilience of millet, the introduction of millet as a backup plant or an additional harvest to complement wheat and barley during challenging environmental circumstances is possible. Although located at some geographic and temporal distance from the present study, a similar argument has been made in the context of the introduction of millet and more diverse land use in northern Italy between the Roman and Late Antiquity and the Medieval periods^90^. Thus, millet could have played a crucial role in sustaining communities during critical times, which might have returned to traditional practices when conditions returned to a cooler and wetter climate.

More local, palaeoenvironmental studies, as well as detailed records of agronomic activities through time, and further archaeological research are required from the Kuckenburg/Esperstedt micro-region, as well as the wider region, to determine the validity of this comparison. Meanwhile, aDNA and strontium isotope approaches can provide more direct insights as to whether the economic and limited burial changes are linked to population movements^91–93^. It is likely that the adoption, utilisation, and eventual abandonment of millet, and its relationship to climatic changes, was mediated by a combination of factors, including climatic shifts, extensive trade networks, and cultural and social influences. Nevertheless, our study provides valuable insights into long-term adaptive plasticity by agricultural societies facing changeable environmental conditions. By studying how past societies responded to environmental changes and climatic threats, we can offer guidance and examples for present-day actions, especially in light of the current challenges posed by climate change and increasing natural disasters^85,94^. Notably, in recognition of climate change challenges, millet is now being acknowledged as an essential component to enhance global food security in various regions worldwide, including Germany^95,96^. More specifically, in Saxony-Anhalt and Bavaria, farmers are experimenting with the cultivation of millet as a supplementary crop, in response to the recent surge in high temperatures during summers^97,98^. Furthermore, in a significant move, the United Nations General Assembly declared 2023 as the International Year of Millets. This declaration aims to draw attention to the nutritional and health benefits of millets, as well as their adaptability for cultivation under adverse and fluctuating climatic conditions. The initiative seeks to promote the sustainable use and cultivation of millets as a key strategy to address food security and nutrition challenges in the face of a changing climate.

## Materials and methods

### Overview

We performed stable carbon (δ^13^C) and nitrogen (δ^15^N) isotope analysis of bone collagen on 53 individuals (3 from the MN, 3 from the FN, 5 from the EBA, and 42 from the LBA) from the two studied sites to reconstruct their dietary practices. Specifically, we analysed 22 individuals from the site of Kuckenburg (i.e., 3 MN and 19 LBA) and 31 individuals from the site of Esperstedt (i.e., 3 FN, 5 EBA and 23 LBA). Stable carbon (δ^13^C_en_) analysis of tooth enamel was also performed on individuals with available teeth (*n* = 33) to better understand the dietary patterns of EBA and LBA individuals. Faunal skeletal remains were also analysed representing cattle, pigs, horses, sheep/goats, and dogs from the same period and context as the human individuals to explore the isotopic variation of dietary sources and whether managed animals had consumed C_4_ plants. Taxonomic identifications of the faunal remains were based on skeletal morphology^99^. We also performed archaeobotanical analysis on five charred broomcorn millet seeds. Finally, we obtained 57 new radiocarbon dates from humans, fauna, and millet seeds.

### Osteoarchaeology

The recommendations of the Society of European Anthropologists served as the basis for estimating the age and sex of the skeletons^100^. Sex determination relied on morphological criteria involving the skull, pelvis, and long bones^101–103^. Age estimation involved assessing surface changes of the *symphysialis ossis pubis*^104–107^, the extent of cranial suture obliteration^108^, tooth abrasion^109–111^, and epiphyseal closure in long bones, clavicle, and pelvis^110,112^. Subadult individuals' ages were estimated based on tooth development and eruption status^103^, as well as the length of their long bones^112,113^. Age classification followed R. Martin's levels^114^. Male skeleton height was estimated using E. Breitinger's formula^115^, while female skeleton height was determined according to H. Bach's method^116^. Dental status was documented using a dental diagram and the corresponding dental code^117,118^, following the Federation Dentaire International system for tooth naming. Under this system, each tooth in both the primary and secondary dentitions is assigned two distinct numbers. The first number designates the quadrant in which the tooth is situated, while the second specifies the precise position of the tooth within that quadrant. Pathological alterations, epigenetic features, and any distinctive features were documented, and comprehensive measurements and indices were collected from the skulls and long bones of adult individuals whenever possible.

### ^14^C dating

Human and animal samples (ca. 1 g of bone) were sent to Curt-Engelhorn-Center Archaeometry gGmbH in Mannheim, Germany for direct radiocarbon dating to establish a chronology for the different dietary patterns. Specifically, 22 individuals from Kuckenburg and 22 individuals from Esperstedt were directly dated. In addition, 8 animals from features of interest were dated to confirm the archaeological assignment to the LBA. Finally, 5 charred broomcorn millet seeds from Kuckenburg were sent to NOSAMS, the National Ocean Sciences Accelerator Mass Spectrometry Facility at the Woods Hole Oceanographic Institution (WHOI) to confirm they belong to the LBA and that they are not a later intrusion caused by the movement of insects or animals. After being washed with water in the archaeobotany lab, the seeds undergo a series of acid–base-acid leaches designed to remove inorganic carbon and/or base-soluble organic acids that may have originated from surrounding sediments prior to combustion and may contaminate the sample with carbon of a different age at the National Ocean Sciences Accelerator Mass Spectrometry Laboratory (after the Sample Preparation Protocol from NOSAMS Facility (https://www2.whoi.edu/site/nosams/resources/methods/).

For the collagen extraction from the bone samples the lab used the modified Longin method, and the collagen was purified by ultrafiltration (fraction > 30kD) and freeze-dried.

The dates were already calibrated when received with OxCal 4.4^119^ using the IntCal 20 curve^120^. The C/N_atomic_ ratios stay within the 2.9–3.6 range indicating good quality collagen^44,46^ (see Supplementary Table 1 (S1) for detailed information of all the dates). All calibrated dates are reported in the text as 95.4% (2σ) probability ranges.

### Archaeobotany

The flotation of samples and the archaeobotanical analysis were conducted at the Archaeobotanik Labor Zach, Bernbeuren, Bavaria. The laboratory provides specialist archaeobotanical services for Germany and beyond and has a comprehensive modern reference collection of Middle European seeds and an extensive library. The flotation technique for archaeobotanical samples followed a standardised protocol in which the material is dissolved and washed in water. Floating organic material is collected in sieves with the minimum mesh width of 0.2 mm. The charred, dried, so-called light fraction is then analysed under a microscope (Leica S9i) with 6 × to 50 × magnification.

### Stable isotope analysis

#### *Diet isotopes—collagen (δ*^*13*^*C and δ*^*15*^*N) and enamel (δ*^*13*^*C)*

In this study, we sampled ribs, unless unavailable, for bulk bone collagen stable isotope analysis to obtain a dietary signal representing roughly the last 10 years of life^121^. For the stable isotope analysis of tooth enamel we sampled premolars, first molars, second molars, and third molars, representing ages from early childhood to adolescence^122^. All steps necessary for these analyses (e.g., collagen extraction, determination of stable carbon and nitrogen isotope ratios, pre-treatment of enamel, stable carbon isotopic composition measurement) took place at the Max Planck Institute of Geoanthropology in Jena, Germany following published protocols^57,123^ (also see Supplementary Text 3: Methods for more details).

### Statistical analyses

We applied Levene's test for the equality of variance in δ^13^C and δ^15^N between time periods at each site, and we used the Shapiro–Wilk test^124^ to assess normality. We performed Kruskal -Wallis tests^48^ to determine if there were significant differences when three or more groups were compared (e.g., different time periods). These tests were chosen to effectively handle the different sample sizes for each time period, to compare the distributions across multiple periods and to account for the non-normality of our data. In order to identify the specific locations of the observed differences among the time periods, we conducted pairwise comparisons using the non-parametric post-hoc Dunn's test^49^, which accounts for the multiple testing issue by applying *p* value adjustments via the Bonferroni correction. We also used correlation tests using the Spearman coefficient for each period to assess if there is a correlation between the δ^13^C and δ^15^N that could reveal the consumption of freshwater or marine resources. A 5% significance level (α = 0.05) was used. All analyses were performed in R 4.2.2^125^.

### Supplementary Information

Supplementary Information 1.

Supplementary Information 2.

## Data Availability

We provide all data reported in this article in the Supplementary Information files. Specifically, radiocarbon dates are listed in Supplementary Table 1 (S1) and the values and quality indicators of the stable isotope analyses (δ^13^C, δ^15^N, δ^13^C_en_) are provided in Supplementary Table 2 (S2). Please refer to Supplementary Table 3 (S3) for a detailed presentation of the results of the statistical tests.

## References

[1] Harding, A. F. *European Societies in the Bronze Age*. (Cambridge University Press, 2000).

[2] Primas, M. Bronzezeit zwischen Elbe und Po. Strukturwandel in Zentraleuropa, 2200–800 v. Chr. *Universitätsforschungen zur prähistorischen Archäologie* **150**, (2008).

[3] Vandkilde H (2016). Bronzization: The Bronze Age as Pre-Modern Globalization. Praehistorische Zeitschrift.

[4] Radivojević M (2019). The provenance, use, and circulation of metals in the European bronze age: The state of debate. J. Archaeol. Res..

[5] Vandkilde, H. Forward. in *Rooted in Movement: Aspects in Mobility in Bronze Age Europe* (eds. Reiter, S., Norgaard, H. W., Kölcze, Z. & Rassmann, C.) 9–10 (Jutland Archaeological Society Publications, 2014).

[6] Cavazzuti C (2022). The first “Urnfields” in the plains of the Danube and the Po. J. World Prehist..

[7] van der Merwe NJ (1982). Carbon Isotopes, Photosynthesis, and Archaeology: Different pathways of photosynthesis cause characteristic changes in carbon isotope ratios that make possible the study of prehistoric human diets. Am. Sci..

[8] Tieszen LL (1991). Natural variations in the carbon isotope values of plants: Implications for archaeology, ecology, and paleoecology. J. Archaeol. Sci..

[9] Pearcy RW, Ehleringer J (1984). Comparative ecophysiology of C3 and C4 plants. Plant Cell Environ..

[10] Porter JR, Gawith M (1999). Temperatures and the growth and development of wheat: A review. Eur. J. Agron..

[11] Asseng S (2014). Rising temperatures reduce global wheat production. Nat. Clim. Chang..

[12] Hunt HV (2008). Millets across Eurasia: Chronology and context of early records of the genera Panicum and Setaria from archaeological sites in the Old World. Veg. Hist. Archaeobot..

[13] Motuzaite-Matuzeviciute G, Hunt HV, Liu X, Jones MK (2013). The early chronology of broomcorn millet (Panicum miliaceum) in Europe. Antiquity.

[14] Filipović D (2020). New AMS 14C dates track the arrival and spread of broomcorn millet cultivation and agricultural change in prehistoric Europe. Sci. Rep..

[15] Liu X (2019). From ecological opportunism to multi-cropping: Mapping food globalisation in prehistory. Quat. Sci. Rev..

[16] Liu, X., G. Motuzaite Matuzeviciute & H.V. Hunt. From a fertile idea to a fertile arc: The origins of broomcorn millet 15 years on. in *Far from the Hearth: Essays in Honour of Martin K. Jones* (eds. E. Lightfoot, X. L. &. D. Q. F.) 155–164 (Cambridge: McDonald Institute Conversations, 2018).

[17] Spengler RN (2015). Agriculture in the Central Asian Bronze Age. Journal of World Prehistory.

[18] Kirleis, W., Corso, M. D. & Filipović, D. *Millet and What Else? The Wider Context of the Adoption of Millet Cultivation in Europe*. (Sidestone Press, 2022).

[19] Hermes TR (2019). Early integration of pastoralism and millet cultivation in Bronze Age Eurasia. Proc. Biol. Sci..

[20] Martin L (2021). The place of millet in food globalization during Late Prehistory as evidenced by new bioarchaeological data from the Caucasus. Sci. Rep..

[21] Smith BN, Epstein S (1971). Two categories of c/c ratios for higher plants. Plant Physiol..

[22] Schwarcz HP, Schoeninger MJ (1991). Stable isotope analyses in human nutritional ecology. Am. J. Phys. Anthropol..

[23] Schoeninger, M. J. Diet reconstruction and ecology using stable isotope ratios. in *A Companion to Biological Anthropology* 445–464 (Wiley-Blackwell, 2010).

[24] van der Merwe NJ, Vogel JC (1978). 13C content of human collagen as a measure of prehistoric diet in woodland North America. Nature.

[25] Schwarcz, H. P., Melbye, J., Anne Katzenberg, M. & Knyf, M. Stable isotopes in human skeletons of Southern Ontario: reconstructing Palaeodiet. *J. Archaeol. Sci.* **12**, 187–206 (1985).

[26] Katzenberg, M. A. Stable isotope analysis: A tool for studying past diet, demography, and life history. in *Biological Anthropology of the Human Skeleton* 411–441 (John Wiley & Sons, Inc., 2008).

[27] Lee-Thorp JA (2008). On isotopes and old bones. Archaeometry.

[28] Ambrose, S. H. & Norr, L. Experimental Evidence for the Relationship of the Carbon Isotope Ratios of Whole Diet and Dietary Protein to Those of Bone Collagen and Carbonate. in Prehistoric Human Bone (eds. Lambert, J.B., Grupe, G.) (Springer, Berlin, Heidelberg, 1993). https://doi.org/10.1007/978-3-662-02894-0_1

[29] Ambrose SH, DeNiro MJ (1986). Reconstruction of African human diet using bone collagen carbon and nitrogen isotope ratios. Nature.

[30] Crawford K, Mcdonald RA, Bearhop S (2008). Applications of stable isotope techniques to the ecology of mammals. Mamm. Rev..

[31] Pospieszny Ł (2021). Isotopic evidence of millet consumption in the Middle Bronze Age of East-Central Europe. J. Archaeol. Sci..

[32] Price TD (2019). Multi-isotope proveniencing of human remains from a Bronze Age battlefield in the Tollense Valley in northeast Germany. Archaeol. Anthropol. Sci..

[33] Reed, K., Kudelić, A., Essert, S., Polonijo, L. & Vrdoljak, S. House of Plenty: Reassessing Food and Farming in Late Bronze Age Croatia. *Environ. Archaeol.* 1–17 (2021).

[34] González-Rabanal B, Marín-Arroyo AB, Cristiani E, Zupancich A, González-Morales MR (2022). The arrival of millets to the Atlantic coast of northern Iberia. Sci. Rep..

[35] Tafuri MA, Craig OE, Canci A (2009). Stable isotope evidence for the consumption of millet and other plants in Bronze Age Italy. Am. J. Phys. Anthropol..

[36] Valamoti SM (2016). Millet, the late comer: on the tracks of Panicum miliaceum in prehistoric Greece. Archaeol. Anthropol. Sci..

[37] Ventresca Miller, A. *et al.* Subsistence and social change in central Eurasia: stable isotope analysis of populations spanning the Bronze Age transition. *J. Archaeol. Sci.* **42**, 525–538 (2014).

[38] Ananyevskaya E (2018). Early indicators to C4 plant consumption in central Kazakhstan during the Final Bronze Age and Early Iron Age based on stable isotope analysis of human and animal bone collagen. Archaeol. Res. Asia.

[39] Ventresca-Miller AR (2023). Adaptability of millets and landscapes: ancient cultivation in north-central Asia. Agronomy.

[40] Hellmund, M. On the “ancient” evidence for Panicum miliaceum and Vicia faba in central Germany (primarily Saxony-Anhalt). in *Millet and what else? The Wider Context of the Adoption of Millet Cultivation in Europe. Scales of Transformations in Prehistoric and Archaic Societies, vol. 14.* (ed. W. Kirleis, M. Dal Corso, D. Filipović) 129–154 (Sidestone Press, 2022).

[41] Lanting JN, Aerts-Bijma AT, van der Plicht J (2001). Dating of cremated bones. Radiocarbon.

[42] Peschel, K. *Thüringen in vor- und frühgeschichtlicher Zeit, *(Beier & Beran, 1994).

[43] Balfanz, K. & Jarecki, H. Jung - und spätbronzezeitliche Sonderbestattungen in Mitteldeutschland. Quellen und Fragestellungen. *Jahresschrift für Mitteldeutsche Vorgeschichte* **88**, 339–378 (2004).

[44] Deniro MJ (1985). Postmortem preservation and alteration of in vivo bone collagen isotope ratios in relation to palaeodietary reconstruction. Nature.

[45] Ambrose SH (1990). Preparation and characterization of bone and tooth collagen for isotopic analysis. J. Archaeol. Sci..

[46] van Klinken, G. J. Bone Collagen Quality Indicators for Palaeodietary and Radiocarbon Measurements. *J. Archaeol. Sci.* (1999).

[47] Le Huray JD, Schutkowski H (2005). Diet and social status during the La Tène period in Bohemia: Carbon and nitrogen stable isotope analysis of bone collagen from Kutná Hora-Karlov and Radovesice. J. Anthropol. Archaeol..

[48] Kruskal WH, Wallis WA (1952). Use of ranks in one-criterion variance analysis. J. Am. Stat. Assoc..

[49] Dunn OJ (1964). Multiple comparisons using rank sums. Technometrics.

[50] Shin JY, O’Connell T, Black S, Hedges R (2004). Differentiating bone Osteonal turnover rates by density fractionation; validation using the bomb ^14^C atmospheric pulse. Radiocarbon.

[51] Hedges REM, Reynard LM (2007). Nitrogen isotopes and the trophic level of humans in archaeology. J. Archaeol. Sci..

[52] Katsimbri, P. The biology of normal bone remodelling. *Eur. J. Cancer Care* **26**, (2017). 10.1111/ecc.12740 28786518

[53] Ingo Feeser, Stefanie Schaefer - Di Maida, Stefan Dreibrodt, Jutta Kneisel, Dragana Filipović. On-site to off-site: A multidisciplinary and multiscale consideration of the 13th to 11th century BCE transformation in northern Germany. in *Millet and What Else? The Wider Context of the Adoption of Millet Cultivation in Europe* (eds. Wiebke Kirleis, M. D. C. &. D. F.) 185–215 (Sidestone Press Academics, 2022).

[54] Pearson JA (2007). New light on early caprine herding strategies from isotope analysis: a case study from Neolithic Anatolia. J. Archaeol. Sci..

[55] Chinique de Armas, Y., Mavridou, A.-M., Garcell Domínguez, J., Hanson, K. & Laffoon, J. Tracking breastfeeding and weaning practices in ancient populations by combining carbon, nitrogen and oxygen stable isotopes from multiple non-adult tissues. *PLoS One* **17**, e0262435 (2022). 10.1371/journal.pone.0262435 PMC8809549 35108296

[56] Dufour E, Bocherens H, Mariotti A (1999). Palaeodietary implications of isotopic variability in Eurasian Lacustrine fish. J. Archaeol. Sci..

[57] Richards MP, Hedges REM (1999). Stable isotope evidence for similarities in the types of marine foods used by late mesolithic humans at sites along the Atlantic coast of Europe. J. Archaeol. Sci..

[58] Guiry EJ (2013). A canine surrogacy approach to human paleodietary bone chemistry: past development and future directions. Archaeol. Anthropol. Sci..

[59] Perri AR, Koster JM, Otárola-Castillo E, Burns JL, Cooper CG (2019). Dietary variation among indigenous Nicaraguan horticulturalists and their dogs: An ethnoarchaeological application of the Canine Surrogacy Approach. J. Anthropol. Archaeol..

[60] Vogel, J. C. Recycling of carbon in a forest environment. *Oecologia Plantarum* 89–94 (1978).

[61] van der Merwe NJ, Medina E (1991). The canopy effect, carbon isotope ratios and foodwebs in amazonia. J. Archaeol. Sci..

[62] Münster A (2018). 4000 years of human dietary evolution in central Germany, from the first farmers to the first elites. PLoS One.

[63] Fenner JN (2005). Cross-cultural estimation of the human generation interval for use in genetics-based population divergence studies. Am. J. Phys. Anthropol..

[64] Hastorf, C. A. *The Social Archaeology of Food: Thinking about Eating from Prehistory to the Present*. (Cambridge University Press, 2016).

[65] Sutton, D. E. *Remembrance of Repasts: An Anthropology of Food and Memory*. (Berg, 2001).

[66] Hamilakis Y (1999). Food technologies/technologies of the body: the social context of wine and oil production and consumption in Bronze Age Crete. World Archaeol..

[67] Hamilakis, Y. The Past as Oral History. in *Thinking through the Body: Archaeologies of Corporeality* (eds. Hamilakis, Y., Pluciennik, M. & Tarlow, S.) 121–136 (Springer US, 2002).

[68] Douglas Price, T. *Europe’s First Farmers*. (Cambridge University Press, 2000).

[69] Shennan, S. *The First Farmers of Europe: An Evolutionary Perspective*. (Cambridge University Press, 2018).

[70] Anthony DW (1990). Migration in Archeology: The baby and the bathwater. Am. Anthropol..

[71] Frachetti MD (2011). Migration concepts in central Eurasian. Archaeology..

[72] Schmidt, B. Die jungbronzezeitlichen Stämme im Elbe-Saale-Gebiet. in *Mitteleuropäische Bronzezeit. Beiträge zur Archäologie und Geschichte* (eds. Coblenz, W. & Horst, F.) 136–212 (1978).

[73] Schmidt B, Nitzschke W (1974). Bestattungssitten der spätbronzezeitlichen Helsmdorfer- und Saalemündungsgruppe. Ausgrab. Funde.

[74] Meller H (2015). Glutgeboren: Mittelbronzezeit bis Eisenzeit. Begleithefte zur Dauerausstellung im Landesmuseum für Vorgeschichte Halle.

[75] Litt T, Schölzel C, Kühl N, Brauer A (2009). Vegetation and climate history in the Westeifel Volcanic Field (Germany) during the past 11 000 years based on annually laminated lacustrine maar sediments. Boreas.

[76] Mayewski PA (2004). Holocene climate variability. Quat. Res..

[77] Kaniewski D, Guiot J, Van Campo E (2015). Drought and societal collapse 3200 years ago in the Eastern Mediterranean: a review. WIREs Climate Change.

[78] Kaniewski, D. & Van Campo, E. 3.2 ka BP Megadrought and the Late Bronze Age Collapse. in Megadrought and Collapse: From Early Agriculture to Angkor, (ed. Harvey Weiss) (New York; online edn, Oxford Academic, 2017) 10.1093/oso/9780199329199.003.0005.

[79] A. Bernard Knapp & Sturt W. Manning. Crisis in Context: The End of the Late Bronze Age in the Eastern Mediterranean. *Am. J. Archaeol.* **120**, 99–149 (2016).

[80] Molloy, B. Was There a 3.2 ka Crisis in Europe? A critical comparison of climatic, environmental, and archaeological evidence for radical change during the bronze age–iron age transition. *J. Archaeol. Res.* (2022) 10.1007/s10814-022-09176-6.

[81] Demény A (2019). Middle Bronze Age humidity and temperature variations, and societal changes in East-Central Europe. Quat. Int..

[82] Regattieri E (2019). Holocene Critical Zone dynamics in an Alpine catchment inferred from a speleothem multiproxy record: disentangling climate and human influences. Sci. Rep..

[83] Molloy B (2023). Resilience, innovation and collapse of settlement networks in later Bronze Age Europe: New survey data from the southern Carpathian Basin. PLoS One.

[84] Dultz, S. Salzanreicherung in Böden aus Löß im Mitteldeutschen Trockengebiet. in *Umweltgeochemie in Wasser, Boden und Luft: Geogener Hintergrund und anthropogene Einflüsse* (eds. Huch, M. & Geldmacher, H.) 3–17 (Springer Berlin Heidelberg, 2001).

[85] Brázdil R (2020). Central Europe, 1531–1540 CE: The driest summer decade of the past five centuries?. Clim. Past.

[86] Camenisch C (2020). Extreme heat and drought in 1473 and their impacts in Europe in the context of the early 1470s. Regional Environ. Change.

[87] Laaha G (2017). The European 2015 drought from a hydrological perspective. Hydrol. Earth Syst. Sci..

[88] Hoy A, Hänsel S, Skalak P, Ustrnul Z, Bochníček O (2017). The extreme European summer of 2015 in a long-term perspective. Int. J. Climatol..

[89] UFZ. Drought development in 2018. *UFZ* (2018). https://www.ufz.de/index.php?de=44429

[90] Riccomi G (2020). Stable isotopic reconstruction of dietary changes across Late Antiquity and the Middle Ages in Tuscany. J. Archaeol. Sci.: Rep..

[91] Haak W (2008). Ancient DNA, Strontium isotopes, and osteological analyses shed light on social and kinship organization of the Later Stone Age. Proc. Natl. Acad. Sci. U.S.A..

[92] Mittnik A (2019). Kinship-based social inequality in Bronze Age Europe. Science.

[93] Knipper C (2017). Female exogamy and gene pool diversification at the transition from the Final Neolithic to the Early Bronze Age in central Europe. Proc. Natl. Acad. Sci. U.S.A..

[94] Ionita, M., Nagavciuc, V., Kumar, R. & Rakovec, O. On the curious case of the recent decade, mid-spring precipitation deficit in central Europe. *NPJ Clim. Atmos. Sci.* **3**, 1–10 (2020).

[95] Saxena R, Vanga SK, Wang J, Orsat V, Raghavan V (2018). Millets for food security in the context of climate change: A review. Sustain. Sci. Pract. Policy.

[96] Karthick K, Arun A, Akshaya V (2023). Proso Millet: Forgotten food for the future. Int. J. Home Sci..

[97] Orzessek Dieter, Deubel Annette, Gille Stefan ,Dallmann J., Schröder J. *Ergebnisse aus den Versuchen zum Anbau von Körnerhirse 2022*. (2023).

[98] LfL. Körnerhirse als neue Kultur in Fruchtfolgesystemen für Trockengebiete. *LfL* https://lfl.bayern.de/koernerhirse.

[99] Schmid, E. *Atlas of animal bones. For prehistorians, archaeologists and Quaternary geologists*. (Elsevier Pub. Co., 1972).

[100] Ferembach, D., Schwidetzky, I. & Stloukal, M. Empfehlungen für die Alters-und Geschlechtsdiagnose am Skelett. (Recommandations pour le diagnostic de l’âge et du sexe sur les squelettes). *Homo Gottingen* **30**, 1–32 (1979).

[101] Phenice TW (1969). A newly developed visual method of sexing the os pubis. Am. J. Phys. Anthropol..

[102] Acsádi, G. & Nemeskéri, J. *History of Human Life Span and Mortality*. (Akadémiai Kiadó, 1970).

[103] Ubelaker, D. H. Human Skeletal Remains. *Excavation, Analysis, Interpretation. *(Taraxacum, 1989).

[104] Todd, T. W. Age Changes in the Pubic Bone. *Am. J. Phys. Anthropol.* **4**, 1-70 (1921).

[105] McKern, T. W., Stewart, T. D. & Quartermaster Research and Engineering Center (U.S.). *Skeletal Age Changes in Young American Males: Analysed from the Standpoint of Age Identification*. (Headquarters, Quartermaster Research & Development Command, 1957).

[106] Katz D, Suchey JM (1986). Age determination of the male os pubis. Am. J. Phys. Anthropol..

[107] Gilbert BM, McKern TW (1973). A method for aging the female Os pubis. Am. J. Phys. Anthropol..

[108] La Vallois HV (1937). durée de la vie chez l’homme fossile. Anthropologie.

[109] Miles, A. E. W. The dentition in the assessment of individual age in skeletal material. in *Dental Anthropology* (ed. Brothwell, D. R.) 191–209 (Pergamon, 1963).

[110] Brothwell, D. R. *Digging Up Bones: The Excavation, Treatment and Study of Human Skeletal Remains*. (British Museum (Natural History), 1972).

[111] Lovejoy CO (1985). Dental wear in the Libben population: its functional pattern and role in the determination of adult skeletal age at death. Am. J. Phys. Anthropol..

[112] Nemeskéri J, Harsányi L, Acsädi G (1960). Methoden zur Diagnose des Lebensalters von Skelettfunden. Anthropol. Anz..

[113] Stloukal, M. & Hanáková, H. Die länge der Längsknochen altslawischer Bevölkerungen unter besonderer Berücksichtigung von Wachstumsfragen. *Homo* **29**, 53–69 (1978).

[114] Martin, R. *Lehrbuch der Anthropologie in systematischer Darstellung: mit besonderer Berücksichtigung der anthropologischen Methoden für Studierende Ärzte und Forschungsreisende*. (Fischer, 1914).

[115] Breitinger E (1937). Zur Berechnung der Körperhöhe aus den langen Gliedmaßenknochen. Anthropol. Anz..

[116] Bach H (1965). Zur Berechnung der Körperhöhe aus den langen Gliedmaßenknochen weiblicher Skelette. Anthropol. Anz..

[117] Bach, A., Ernst, G., Finke, L. & Hohmann, G. Germanen, Slawen, Deutsche. Anthropologische Bearbeitung des frühmittelalterlichen Gräberfeldes von Rohnstedt, Kreis Sondershausen. *Germanen, Slawen, Deutsche. Anthropologische Bearbeitung des frühmittelalterlichen Gräberfeldes von Rohnstedt, Kreis Sondershausen *(1986).

[118] Bach, H. & Bach, A. Paläanthropologie im Mittelelbe-Saale-Werra-Gebiet : Beiträge zur Rekonstruktion der biologischen Situation ur- und frühgeschichtlicher Bevölkerungen. in *Weimarer Monographien zur Ur- und Frühgeschichte* vol. 23 (Museum für Ur- und Frühgeschichte Thüringens, 1989).

[119] Ramsey CB (2009). Bayesian analysis of radiocarbon dates. Radiocarbon.

[120] Reimer PJ (2020). The IntCal20 northern hemisphere radiocarbon age calibration curve (0–55 cal kBP). Radiocarbon.

[121] Meier-Augenstein W (2017). Stable Isotope Forensics: Methods and Forensic Applications of Stable Isotope Analysis.

[122] Nelson, S. J. *Wheeler’s Dental Anatomy, Physiology and Occlusion - E-Book: Wheeler's Dental Anatomy, Physiology and Occlusion - E-Book*. (Elsevier Health Sciences, 2014).

[123] Longin R (1971). New method of collagen extraction for radiocarbon dating. Nature.

[124] Shapiro SS, Wilk MB (1965). An analysis of variance test for normality (Complete Samples). Biometrika.

[125] R Core Team (2021). R: A language and environment for statistical computing. R Foundation for Statistical Computing, Vienna, Austria. *The R Project for Statistical Computing* https://www.R-project.org/.

