# Supplementary material for: Biomolecular evidence for changing millet reliance in Late Bronze Age central Germany
Source: Sci Rep. 2024 Feb 22;14:4382. doi: 10.1038/s41598-024-54824-0 (PMC10883991; doi:10.1038/s41598-024-54824-0)
Supplement: Supplementary file 2 — Supplementary Information 2. [file 41598_2024_54824_MOESM2_ESM.docx]

**Supplementary Information for**

**Biomolecular evidence for changing millet reliance in Late Bronze Age central Germany**

**Authors:** Eleftheria Orfanou^1,2,3,*^, Barbara Zach^2,3,9^, Adam B. Rohrlach^1,4^, Florian N. Schneider^3^, Enrico Paust^3^, Mary Lucas^2,5^, Taylor Hermes^1,6^, Jana Ilgner^2^, Erin Scott^2^, Peter Ettel^3^, Wolfgang Haak^1^, Robert Spengler^2,9^, Patrick Roberts^2,7,8,*^

^1^Department of Archaeogenetics, Max Planck Institute for Evolutionary Anthropology, 04103 Leipzig, Germany

^2^Department of Archaeology, Max Planck Institute of Geoanthropology, 07745 Jena, Germany

^3^Chair of Pre- and Protohistoric Archaeology, Friedrich-Schiller University Jena, 07743 Jena, Germany

^4^School of Computer and Mathematical Sciences, University of Adelaide, 5005 Adelaide, Australia

^5^Arctic University Museum of Norway, UiT-the Arctic University of Norway, Lars Thørings veg 10, 9006 Tromsø, Norway

^6^ Department of Anthropology, University of Arkansas, 72701 Fayetteville, United States of America

^7^isoTROPIC Research Group, Max Planck Institute of Geoanthropology, 07745 Jena, Germany

^8^Institut für Ur- und Frühgeschichte, Philosophische Facultät, Universität zu Köln, Cologne, Germany

^9^Domestication and Anthropogenic Evolution Research Group, Max Planck Institute of Geoanthropology, 07745 Jena, Germany

*Corresponding authors: [eleftheria_orfanou@eva.mpg.de](mailto:eleftheria_orfanou@eva.mpg.de); [roberts@gea.mpg.de](mailto:roberts@shh.mpg.de)

**This PDF file includes:**

Supplementary Text 1: Kuckenburg

Supplementary Text 2: Esperstedt

Supplementary Text 3: Methods

Supplementary references

## **Supplementary Text 1: Kuckenburg**

Kuckenburg is a Late Bronze Age (LBA) fortified hilltop site located on top of the Kranzberg spur, surrounded by the Weida stream, in Saxony-Anhalt, central Germany. Despite the discovery of a LBA hoard on the Kranzberg in 1901/1902 ^1,2^, systematic archaeological research only began in 2005, with excavations directed by the Chair of Prehistory and Early History at the Friedrich-Schiller-Universität Jena in 2005, 2007, 2009-2012 and annually since 2015 ^3^. The excavations since 2005 have been supplemented by an extensive geophysical survey of the site. Archaeologically, 22 separate areas totalling 3,290 m² have been excavated so far. Most LBA occupation areas are located directly on the spur, which was divided from the hinterland by a ditch. The excavations of 2020 and 2021 focused on areas beyond the ditch for the first time. In 2021, in this context, pit clusters were targeted for the first time; these pits cover the Kranzberg according to the results of the geophysical study.

As part of these excavations, LBA settlement features have been documented across the entire area, between the tip of the spur and the eastern forefield of the LBA ditch. The geophysical prospections on the Kranzberg show a scatter of features, stretching almost as far as the route of the federal motorway (Bundesautobahn) BAB 38. However, as the site was also used as a settlement in the late 4th millennium BCE, as well as in the Early Middle Ages, the scatter of features cannot be equated with the extent of the LBA settlement, unless test trenches are excavated. Nevertheless, it seems probable that the settlement extended no farther than the BAB 38, since no LBA features were documented there. In this entire area, the spatial distribution of the available ^14^C dates and the archaeological record indicate two LBA settlement phases: an older one from the early LBA on the eastern forefield of the LBA ditch; and a younger one mainly from the 10^th^ and 9^th^ centuries BCE, directly on the spur.

In addition, 43 human graves have been identified on the hillfort settlement of Kuckenburg. As the ^14^C dates show, most of them stretch back to the 10^th^ and especially the 9^th^ centuries BCE, with one individual from the 13^th^ century BCE. The individuals were deposited both individually and in groups, in settlement pits and ditches, within a fortified sub-area of the settlement on the spur of Kuckenburg.

## **Supplementary Text 2: Esperstedt**

Esperstedt is located on the opposite side of the stream Weida, a few hundred meters away from Kuckenburg, and it consists of a LBA settlement and a graveyard (Fig.1). The settlement west of the Weida is clearly separated from the adjacent burial ground on the plateau by a ditch. The areas of the settlement and the burial ground thus experience a clear functional distinction. The LBA occupants of the site presumably belonged to the Unstrut group (1325-750 BCE**)**, named after the Unstrut River, a tributary of the Saale. The Unstrut group is distributed mainly in the Thuringian Basin (“Thüringer Becken”) and the southern part of Saxony-Anhalt. Elements defining this group include characteristic ceramic forms and inhumations, which were deposited in so called Steinpackungsgräbern (graves with massive stone build walls). However, around 1000 BCE, cremations became gradually more frequent.

###

### **Settlement**

The settlement area from Esperstedt, like the graveyard, was excavated over a large area, directly before the construction of the BAB 38, after the sporadic uncovering of individual finds in the 20th century. At the same time, geophysical prospection was carried out by the Schweitzer company on a total area of 4,800 m². Settlement features were detected across an area of 25,300 m², with boundaries of the settlement area in the west-northwest (bounded by a ditch) and east-southeast and northeast (boundaries of the feature scatter and bounded by a second ditch, respectively). In addition, in the middle of the of excavation area, a conspicuously feature-poor zone indicates an internal division of the settled area: while one area is mainly located on the plateau of Esperstedt, stretching up the steep slope to the Weidabach valley, the second settlement area extends mainly on the "saddle" of Esperstedt, a sloping tongue of land extending from the plateau to the northeast. Compared to the first settlement area, which lies at an altitude of 181-171 m above sea level, this settlement area is situated considerably lower (i.e., 170-148 m above sea level).

The original settled area, however, was considerably larger than the area covered by the excavation. This is shown by the fact that not all boundaries of the settlement area were recorded during the 2004 excavations (especially in the north and south). In addition, the results of the geophysical prospection, as well as the distribution of old finds, speak to the likelihood of a further extension of the settlement. For example, the geophysical prospection of 2004 shows both an extension of one ditch to the southwest, which is likely to have delimited the Esperstedt plateau towards Weidabach valley, and a scatter of features from settlement pits lying beyond the 2004 excavation boundaries on the plateau between this ditch and the steep slope towards Weidabach valley. Against this background, it can be assumed that the higher lying settlement area was probably three to four times larger than the excavation area of 2004. The discovery of a settlement pit in 1962 directly south of the present route of the BAB 38 also fits this picture. Furthermore, in the 1990s, settlement pits were discovered during ploughing on the "saddle", in a larger area located directly northwest of the excavation area^3^. Therefore, it is likely that the entire saddle was occupied during the Late Bronze Age. Additionally, taking into account the terrain topography, the original extent of the second settlement area would have been approximately 8-10 times larger than excavated area.

There are depositions of 87 individuals in Esperstedt. These depositions are neither concentrated in a structurally defined quarter of the settlement nor do they exhibit a density comparable to the Kuckenburg settlement. Rather, the depositions are scattered over the entire archaeological site, which is many times larger than Kuckenburg. In addition, the available ^14^C dates suggest a dating from the 12^th^ to 9^th^ centuries BCE. However, an assessment of this chronological contrast between the two settlement sites is not possible on the basis of the current state of research, because the sample size is too small and the standard deviations are about 100 years and, thus, are twice as high as those for the dates of Kuckenburg.

At both sites many individuals were laid down in the pits according to “regular” burial customs. However, in some cases the individuals were no longer intact or deposited using practices entirely different from those known from the graveyards (e.g., deposition of single skulls). In other cases, only long bones found their way into the pit fillings. In addition, in 18 pits cremated human remains were found.

### **Graveyard**

After the discovery of the first graves in 1910/1911 ^7^ and 1972 ^2^, large-scale excavations were carried out in 2004 by the LDA of Saxony-Anhalt before the construction of the BAB 38 ^8^. Although a large-scale excavation, it was only partially possible to document the original boundaries of the graveyard, namely in the west, east and northwest. The 2018 excavation, at the commuter car park immediately to the south, also revealed that the southwestern boundary may also have been approximately reached in 2004, as no further LBA burial features were uncovered. To the south and northeast in particular, however, the graveyard extends beyond the 2004 excavation area. This is also indicated by the results of the geophysical prospection of 2004 on an area of 17,600 m² by the Schweitzer company. However, any indications of the original extent of the graveyard are missing.

With 175 graves dating back to the Late Bronze Age, this graveyard is one of the largest in the Unstrut group. The mortuary rites observed here predominantly align with the practices observed within this group, characterized by a prevalence of inhumation burials, the extended supine position in a NW–SE orientation, and the use of stone packs. In some respects, though, the Esperstedt graveyard differs from the published state of the art, notably that many graves exhibit evidence of secondary disturbances such as robber shafts, green discolouration on the bones (suggesting the prior presence of bronze artifacts), and skeletons found in disturbed positions. Considering the special treatment of isolated body parts in the micro-region, this is a most interesting finding because the phenomenon of secondary grave manipulations has not gained much attention in the context of the Late Bronze Age so far ^9^.

The absence of stone cists in the graveyard is noteworthy. This observation is particularly striking considering that stone cists are a distinctive feature of the nearby Saalemündungsgruppe (SMG), and there is even a single known stone cist from Kuckenburg.

However, the presence of 15 cremations at the Esperstedt graveyard points to a possible ritual influence from the SMG. All taken together, this offers the unique opportunity to study the transition zone between societies characterised by fundamentally different mortuary rites.

The internal structure of the graveyard is dominated by at least 23 circular ditches. These were obviously topographical and ritual reference points, as in many cases clusters of graves were constructed around them – possibly from families. Similar features are known from other sites in the vicinity ^10,11^, though there are no in-depth analyses. An urgent desideratum for the Esperstedt graveyard is the ^14^C dating of a representative sample of graves. Currently, absolute dates are only available for 26 LBA graves, which are hardly representative of the graveyard. Furthermore, 14 of these ^14^C dates have a standard deviation of about 100 years. Whether the prior dating of the graveyard to 1400–1000 BCE is reliable is, therefore, as hard to determine as is the chronological relationship to the other mortuary areas in the micro-region.

## **Supplementary Text 3: Methods**

### **Archaeobotanical analyses**

Five selected sediment samples of volumes between 1.8 and 5.7 liters were processed following the standard bucket flotation method. After sitting submerged in water for about 24 hours, the sediment had separated into: 1) Light Fraction, composed of floating organic specimens; and 2) Heavy Fraction, composed of heavy mineralized sediment and rocks. The floating organic material was decanted over three sieves of mesh sizes: 2.5mm, 1.0mm and 0.2mm. Bucket flotation separates out two fractions from a soil sample. The light fraction or flot made up of light, floating organics that floats on top the water and is collected by the small mesh sieves. The sediment made up of fine grains suspended in water flows through the sieves. The heavy fraction made up of stones, rocks, pebbles, and heavy archaeological materials stays in the bucket. The process of adding water and decanting is repeated until the water runs clear and all the light, floating organics are collected by the sieves and the heavy, mainly mineral part of the samples, no longer has any fine material. The light organic residues were fully dried before being analysed, since all archaeological components in them were charred. The seeds then were analysed under a binocular microscope with a magnification of up to 50 times, using a comprehensive reference collection of modern seeds and fruits as well as relevant scientific literature (e.g., ^12^).

### **Stable isotope analyses**

Stable isotope analysis has long been used in archaeology to reconstruct past human and animal diets ^13,14^. δ^13^C and δ^15^N ratios in bone collagen reflect ratios of the diet ^15,16^. These stable isotope values allow us to distinguish among reliance on different sources of food, for example different types of plants (i.e., C_3_, C_4_, CAM) and terrestrial, marine or freshwater resources ^17,18^. δ^13^C and δ^15^N of bone collagen will be biased towards the protein portion of the diet, while δ^13^C of tooth enamel represents the whole diet including carbohydrates and fats ^15^.

δ^13^C variability is largely based on the two dominant photosynthetic pathways of plants, C_3_ and C_4_, differing in their net discrimination against ^13^C during CO_2_ fixation ^19^*.* C_3_ plants (e.g., wheat, barley and most temperate grasses) demonstrate lower δ^13^C values than C_4_ plants (e.g., maize, millet, and most tropical grasses), since they discriminate more against ^13^C during CO_2_, leading to distinct and non-overlapping δ^13^C values. Specifically, δ^13^C values of C_3_ plants vary from about −35 to −20‰ ^14,19^, while δ^13^C values of C_4_ plants range from approximately −14 to −9‰ [^20,21^](https://paperpile.com/c/a10lyC/ahfQp+TENTe). These differences in δ^13^C values are reflected in the tissues of consumers, but with a trophic effect of 1-2‰ between food and bone collagen allowing some estimation of the importance of these different resources in the food chain ^15,22^.

δ^15^N variability on the other hand, is based on the trophic level that an organism belongs to. A stepwise enrichment in ^15^N of about +2-6‰ from plants to herbivores, and from herbivores to carnivores, is well documented in both marine and terrestrial systems ^23,24^. Since marine food chains are longer than terrestrial ones, they demonstrate distinctively high δ^15^N ^25,26^. They also demonstrate higher δ^13^C values than C_3_ ecosystems since they obtain their CO_2_ from bicarbonate sources in the ocean. Freshwater foods also tend to have high δ^15^N though δ^13^C does not follow the same trend towards higher measurements as in marine food chains ^27^. Analysis of human bone collagen should thus reveal human reliance on C_3_ or C_4_ plants (or crops), meat, or marine and freshwater resources. Both δ^13^C and δ^15^N in European temperate ecosystems can be impacted by climatic factors such as aridity, temperature, forest cover and anthropogenic land use ^28–30^. This makes it particularly important to develop ‘baseline’ datasets of animals from the same sites or contexts ^31^.

In this study, ribs were sampled for bone collagen isotope analysis, when possible, to obtain a dietary signal for roughly the last 10 years of life ^32^. The outer surface of the bones was first cleaned either with a drill or by abrasion from a sandblaster. Then samples of up to 1g of bone (i.e., ~ 600 to 1 g) were collected by using a diamond cutting blade to cut the bones into smaller bone chunks, roughly equal in size. Collagen was then extracted following standard procedures ^33^.The samples were demineralized in 10ml aliquots of 0.5M HCL at 4°C*,* with changes of the acid every 48 hours until fully demineralised*.* The demineralisation process can take from 1 to 3 weeks, depending on the preservation of the bone. After the demineralisation step, the sample was rinsed three times with ultra-pure H_2_O to remove acid traces and was then immersed in pH3 HCl solution and placed on a heat block at 70 °C for 48 hours, to gelatinise the sample. The samples were then Ezee-filtered to remove insoluble particles ^34^ and the final solution was then freeze dried for 48 h, or until fully dry. Around 0.3 to 0.6 mg of the resulting purified collagen was weighed in duplicate into tin capsules for bulk isotopic analysis*.*

The stable carbon and nitrogen isotope ratios were determined using a Thermo Scientific Flash 2000 Elemental Analyser connected to a Thermo Delta V Advantage mass spectrometer. All measurements are reported as the ratio of the heavier isotope to the lighter isotope (^13^C/^12^C or ^15^ N/^14^N) in δ ‘permil’ notation (‰) relative to the international standards of VPDB and atmospheric N2 (AIR) for δ^13^C and δ^15^N, respectively. Carbon and nitrogen isotope values were compared, using a two-point calibration, against international standards with known isotopic composition (IAEA-CH-6 Sucrose, IAEAN- 2 Ammonium Sulphate and USGS40 L-Glutamic Acid); USGS40 δ^13^C = − 26.39 ± 0.04‰, δ^15^N = − 4.5 ± 0.1‰; IAEA N2 δ^15^N = 20.41 ± 0.12‰; IAEA C6 δ^13^C = − 10.8 ± 0.47‰. A Sigma fish gel standard (δ^13^C = -15.8 ±0.2‰; δ^15^N = 13.75 ±0.1‰) was run as an in-house standard. Replicate analysis of the standards suggests that machine measurement error was c. ± 0.1‰ for δ^13^C and ± 0.3‰ for δ^15^N. In order to assess collagen preservation, two criteria were used, the atomic C:N ratio and the calculated collagen yield. Samples with collagen yields over 1wt% and C:N ratios between 2.9 and 3.6 were accepted ^35,36^.

We also performed stable isotope analyses of tooth enamel (δ^13^Cen) for individuals from the two sites that had suitable teeth available. Specifically, we sampled first molars (enamel mineralisation between birth and 3 years), premolars (enamel mineralisation between 1.5 and 7 years), second molars (enamel mineralisation between 2.5 and 8 years), and third molars (enamel mineralisation between 7 and 16 years), representing ages from early childhood to adolescence ^37^. The selected cleaned surface of the teeth (i.e., occlusal surface to get a long-term bulk signal) was vertically drilled using a tungsten drill to obtain 6-10 mg of enamel powder. Sample pre-treatment included immersion in 1 mL of 1% NaClO for 60 min, rinsing three times with MilliQ water to remove any remaining bleach, immersion in 0.1 M acetic acid for 10 min, and rinsing three times with MilliQ water to remove all traces of remaining acid. Then the samples were covered with parafilm, frozen, and freeze dried for 4h. From each sample, approximately 3 mg enamel powder was weighed into 12 mL borosilicate glass vials.

Samples were then flush/filled with helium at 100 mL/min for 10 min. Following reaction with 100% phosphoric acid, the gases evolved from the samples were analysed for stable carbon isotopic composition using a Thermo Gas Bench 2 connected to a Thermo Delta V Advantage Mass Spectrometer at the Department of Archaeology, Max Planck Institute of Geoanthropology (formerly Science of Human History). Carbon isotope (δ^13^C) values were calibrated using a two-point calibration against international standards (IAEA NBS 18, IAEA 603, IAEA CO8) registered by the International Atomic Energy Agency: IAEA NBS 18: δ^13^C − 5.014 ± 0.032‰; IAEA 603: δ^13^C + 2.46 ± 0.01‰; IAEA CO8: δ^13^C − 5.764 ± 0.032‰, and USGS44: δ^13^C = ~ − 42.1‰. Replicate analyses of standards suggest that machine measurement error is c. ± 0.1‰ for δ^13^C. Overall measurement precision was studied through the measurement of repeat extracts from a bovid tooth enamel standard (n = 20, ± 0.2‰ for δ^13^C).

## **Supplementary references**

1. Förtsch, O. Depotfund der jüngeren Bronzezeit vom Kranzberge bei Kuckenburg, Kreis Querfurt. *Jahresschrift für die Vorgeschichte der sächsisch-thüringischen Länder* **3**, 3–42 (1904).

2. Wagner, K. Zu Regionalbildung während der Jung- und Spätbronzezeit im Saale-Unstrut-Gebiet. Unpublished thesis (Halle, 1989).

3. Ettel, P. *et al*. Vorbericht zu den Grabungen 2005 bis 2011 der Friedrich-Schiller-Universität Jena auf der Kuckenburg bei Esperstedt, Saalekreis. *Archäologie in Sachsen-Anhalt* [**8**](http://paperpile.com/b/a10lyC/nX5O), 19–38 (2016).

4. Glaser, H. U. & Döhle, H.-J. Eine Siedlung der späten Bronzezeit und frühen Eisenzeit bei Esperstedt. in *Archäologie auf der Überholspur. Ausgrabungen an der A38. Archäologie in Sachsen-Anhalt – Sonderband* **5** (ed. Meller, H.) 133–147 (2006).

5. LDA. *LDA Halle, OA Esperstedt (Nr. 12/50/2)*[.](http://paperpile.com/b/a10lyC/vXYFN)

6. Müller, U. Zufall – Absicht – Abfall? Siedlungsbestattungen im bronzezeitlichen Esperstedt. in *Archäologie auf der Überholspur. Ausgrabungen an der A38. Archäologie in Sachsen-Anhalt – Sonderband* **5** (ed. Meller, H.) 148–159 (2006).

7. Reuß, K. Gräber bei Ober-Esperstedt (Mansfelder Seekreis). *Jahresschrift für Vorgeschichte der sächs.-thür. Länder,* **10**, 17–19 (2011).

8. Grothe, A. & Bogen, C. Das spätbronzezeitliche Gräberfeld von Esperstedt. in *Archäologie auf der Überholspur. Ausgrabungen an der A38. Archäologie in Sachsen-Anhalt – Sonderband* **5** (ed. Meller, H.) 160–194 (2006).

9. Kümmel, C. Ur- und frühgeschichtlicher Grabraub: archäologische Interpretation und kulturanthropologische Erklärung. *Tübinger Schriften zur ur- und frühgeschichtlichen Archäologie* [**9**](http://paperpile.com/b/a10lyC/5r4s), (2009).

10. Moser, A. & Bogen, C. Ein Kreisgrabenfeld der Bronzezeit bei Oechlitz, Saalekreis. In: H. Meller/ M. Becker (Hrsg.). in *Neue Gleise auf alten Wegen II. Jüdendorf bis Gröbers Band II. Archäologie in Sachsen-Anhalt Sonderband* **26/II** (eds. Meller, H. & Becker, M.) 377–387 (2017).

11. Moser, A., Duchniewski, B. & Viol, P. Kreisgräben der Mittel- bis Jungbronzezeit. in *Salzmünde-Schiepzig – ein Ort, zwei Kulturen. Ausgrabungen an der Westumfahrung Halle (A 143) Teil II* (eds. Meller, H. & Friedrich, S.) 169–190. (2019).

12. René T. J. Cappers, Renée M. Bekker, Judith E. A. Jans. *Digitale zadenatlas van Nederland / Digital seed atlas of the Netherlands | 2e Editie*. (Barkhuis Publishing, 2012).

13. Vogel, J. C. & van der Merwe, N. J. Isotopic Evidence for Early Maize Cultivation in New York State. [*Am. Antiq.* **42**](http://paperpile.com/b/a10lyC/FkeQw), 238–242 (1977).

14. van der Merwe, N. J. & Vogel, J. C. 13C content of human collagen as a measure of prehistoric diet in woodland North America. [*Nature*](http://paperpile.com/b/a10lyC/O2A2z) **276**, 815–816 (1978).

15. Ambrose, S. H. & Norr, L. Experimental Evidence for the Relationship of the Carbon Isotope Ratios of Whole Diet and Dietary Protein to Those of Bone Collagen and Carbonate. in Prehistoric Human Bone (eds. Lambert, J.B., Grupe, G.) (Springer, Berlin, Heidelberg, 1993).

16. Schoeninger, M. J. Diet reconstruction and ecology using stable isotope ratios. in *A Companion to Biological Anthropology* 445–464 (Wiley-Blackwell, 2010).

17. Makarewicz, C. A. & Sealy, J. Dietary reconstruction, mobility, and the analysis of ancient skeletal tissues: Expanding the prospects of stable isotope research in archaeology. [*J. Archaeol. Sci.* **56**, 146–158 (2015).](http://paperpile.com/b/a10lyC/Njb77)

18. DeNiro, M. J. & Epstein, S. Influence of diet on the distribution of carbon isotopes in animals. *Geochim. Cosmochim. Acta* **42**, Issue 5, 495-506 (1978).

19. Smith, B. N. & Epstein, S. Two categories of c/c ratios for higher plants. [*Plant Physiol.* **47**](http://paperpile.com/b/a10lyC/XRNt3), 380–384 (1971).

20. Schwarcz, H. P., Melbye, J., Anne Katzenberg, M. & Knyf, M. Stable isotopes in human skeletons of Southern Ontario: reconstructing Palaeodiet. *J. Archaeol. Sci.* [**12**](http://paperpile.com/b/a10lyC/ahfQp), 187–206 (1985).

21. Katzenberg, M. A. Stable isotope analysis: A tool for studying past diet, demography, and life history. in *Biological Anthropology of the Human Skeleton* 411–441 (John Wiley & Sons, Inc., 2008).

22. Lee-Thorp, J. A. On isotopes and old bones. *Archaeometry***50**, 925–950 (2008).

23. DeNiro, M. J. & Epstein, S. Influence of diet on the distribution of nitrogen isotopes in animals. *Geochim. Cosmochim. Acta* **45**, 341-351 (1981).

24. Sealy, J. C., van der Merwe, N. J., Thorp, J. A. L. & Lanham, J. L. Nitrogen isotopic ecology in southern Africa: Implications for environmental and dietary tracing. [*Geochim. Cosmochim. Acta* **51**](http://paperpile.com/b/a10lyC/xdgbm), 2707–2717 (1987).

25. Minagawa, M. & Wada, E. Stepwise enrichment of 15N along food chains: Further evidence and the relation between δ15N and animal age. *Geochim. Cosmochim. Acta* [**48**](http://paperpile.com/b/a10lyC/Up6tC), 1135–1140 (1984).

26. Schoeninger, M. J. & DeNiro, M. J. Nitrogen and carbon isotopic composition of bone collagen from marine and terrestrial animals. *Geochim. Cosmochim. Acta* [**48**](http://paperpile.com/b/a10lyC/4Aqy3), 625–639 (1984).

27. Dufour, E., Bocherens, H. & Mariotti, A. Palaeodietary Implications of Isotopic Variability in Eurasian Lacustrine Fish. [*J. Archaeol. Sci.* **26**](http://paperpile.com/b/a10lyC/P9byt), 617–627 (1999).

28. Ambrose, S. Isotopic analysis of paleodiets: methodological and interpretive considerations. (1993).

29. Schwarcz, H. P., Dupras, T. L. & Fairgrieve, S. I. 15N Enrichment in the Sahara: In Search of a Global Relationship. [*J. Archaeol. Sci.* **26**](http://paperpile.com/b/a10lyC/ddTQ5), 629–636 (1999).

30. Ambrose, S. H. & DeNiro, M. J. The isotopic ecology of East African mammals. [*Oecologia* **69**](http://paperpile.com/b/a10lyC/qO6F3), 395–406 (1986).

31. Bownes, J., Clarke, L. & Buckberry, J. The importance of animal baselines: Using isotope analysis to compare diet in a British medieval hospital and lay population. [*Journal of Archaeological Science: Reports* **17**](http://paperpile.com/b/a10lyC/ezKqG), 103–110 (2018).

32. Meier-Augenstein, W. *Stable Isotope Forensics: Methods and Forensic Applications of Stable Isotope Analysis*. (Wiley & Sons, Limited, John, 2017).

33. Richards, M. P. & Hedges, R. E. M. Stable Isotope Evidence for Similarities in the Types of Marine Foods Used by Late Mesolithic Humans at Sites Along the Atlantic Coast of Europe. [*J. Archaeol. Sci.* **26**](http://paperpile.com/b/a10lyC/CqeHm), 717–722 (1999).

34. Brock, F., Geoghegan, V., Thomas, B., Jurkschat, K. & Higham, T. F. G. Analysis of Bone ‘Collagen’ Extraction Products for Radiocarbon Dating. [*Radiocarbon* **55**](http://paperpile.com/b/a10lyC/3Q9WL), 445–463 (2013).

35. Deniro, M. J. Postmortem preservation and alteration of in vivo bone collagen isotope ratios in relation to paiaeodietary reconstruction. *Nature* **317**, 7–10 (1985).

36. Van Klinken, G. J. Bone collagen quality indicators for palaeodietary and radiocarbon measurements. [*J. Archaeol. Sci.* **26**](http://paperpile.com/b/a10lyC/KZbMW), 687–695 (1999).

37. Nelson, S. J. *Wheeler’s Dental Anatomy, Physiology and Occlusion - E-Book: Wheeler's Dental Anatomy, Physiology and Occlusion - E-Book*. (Elsevier Health Sciences, 2014).
